# Application of Compensation Algorithms to Control the Speed and Course of a Four-Wheeled Mobile Robot

**DOI:** 10.3390/s24227233

**Published:** 2024-11-12

**Authors:** Gennady Shadrin, Alexander Krasavin, Gaukhar Nazenova, Assel Kussaiyn-Murat, Albina Kadyroldina, Tamás Haidegger, Darya Alontseva

**Affiliations:** 1 School of Digital Technologies and Artificial Intelligence, D. Serikbayev East Kazakhstan Technical University, 19 Serikbayev Street, Ust-Kamenogorsk 070010, Kazakhstan; 2 University Research and Innovation Center (EKIK), Óbuda University, Bécsi út 96/b, III. em., 1034 Budapest, Hungary

**Keywords:** wheeled mobile robot (WMR), control algorithm, disturbance compensation, inverse model, reference filter

## Abstract

This article presents a tuned control algorithm for the speed and course of a four-wheeled automobile-type robot as a single nonlinear object, developed by the analytical approach of compensation for the object’s dynamics and additive effects. The method is based on assessment of external effects and as a result new, advanced feedback features may appear in the control system. This approach ensures automatic movement of the object with accuracy up to a given reference filter, which is important for stable and accurate control under various conditions. In the process of the synthesis control algorithm, an inverse mathematical model of the robot was built, and reference filters were developed for a closed-loop control system through external effect channels, providing the possibility of physical implementation of the control algorithm and compensation of external effects through feedback. This combined approach allows us to take into account various effects on the robot and ensure its stable control. The developed algorithm provides control of the robot both when moving forward and backward, which expands the capabilities of maneuvering and planning motion trajectories and is especially important for robots working in confined spaces or requiring precise movement into various directions. The efficiency of the algorithm is demonstrated using a computer simulation of a closed-loop control system under various external effects. It is planned to further develop a digital algorithm for implementation on an onboard microcontroller, in order to use the new algorithm in the overall motion control system of a four-wheeled mobile robot.

## 1. Introduction

With rapid progress in the development of technology, modern society is increasingly introducing robotics into various spheres of life to ensure a high level of safety and comfort [1,2]. Especially since the COVID-19 period, the threshold to enter the market of mobile robotics has lowered, integrating advanced mechatronic design, modularity, 3D printing, and human-centered control approaches [3,4,5]. In modern factory warehouses and production lines, one can see the widespread use of transport robots, which replace traditional human labor and deliver people from the need to perform monotonous and difficult tasks [6]. These “smart” robots are able to effectively perform their functions, subject to the requirements of movement along a given trajectory [7]. Intelligent mobile robots, designed to disengage people from repetitive, time-consuming, or dangerous tasks, are attracting attention from both the business and scientific communities due to their potential broad industrial applications [8,9] and the variety of scientific methods and approaches to their control.

The design of wheeled robot motion control systems traditionally focuses on kinematic parameters. For example, Zhang et al. (2024) developed a kinematic model of a four-wheeled robot that takes into account the slipping effect. An adaptive kinematic control algorithm based on slip estimation according to Lyapunov’s theory was designed for uncertain inclined planes [10]. However, to improve the accuracy of following given trajectories, it is necessary to include dynamic characteristics into the calculations, especially when developing robots to move quickly or carry heavy loads. This will significantly reduce tracking errors and improve the overall performance of robotic systems [11].

A mobile robot is a dynamic nonlinear system that takes advantage of the capabilities of autonomous control and various control methods [12].

In this research, the emphasis is on a wheeled mobile robot, considered as a control object, i.e., the plant within the traditional control loop. Such a robot, known as WMR (wheeled mobile robot), is a transport device with motors that ensure the ability to move independently along a given trajectory [13]. In modern scientific and industrial environments, WMRs are of significant interest due to their functionalities, applications, adaptability, and integration with modern technologies and systems [14].

Currently, a significant amount of research is being carried out on control systems for mobile robots. It should be noted that the synthesis of control algorithms for wheeled robots mainly uses neural network algorithms, sliding-mode control, and model-based control methods. Each of these methods, along with certain advantages, also has some drawbacks. For example, neural network algorithms for wheeled robots are characterized by lengthy development and training processes, a significant amount of controller memory usage, and the requirement for special hardware (such as GPU processors) [15,16,17].

Sliding-mode control techniques involve discontinuous control and the occurrence of high-frequency oscillations in the control actions within the control system [10,13], which reduces the reliability of the power part of the system and slows down the movement towards the desired trajectory.

The model-based control method [18,19] only allows for the correction of the control object’s own dynamics, but it would also be desirable to ensure compensation of external disturbances with a certain accuracy.

Robot control research is an active area where the development of new algorithms plays a key role in improving the performance and reliability of robotic systems.

Developing effective control strategies for wheeled mobile robots requires taking into account the complexity and multivariance of their mathematical models, as well as the use of modern control and optimization methods to ensure stable and accurate movement under various conditions.

### Model-Based Control for Disturbance Rejection

In automatic control theory, there is a common problem with constructing an accurate model of the control object. This means the impossibility of accounting for the entire set of factors affecting the control object, the complexity of modeling certain factors such as skidding in wheeled vehicles, and such uncertainties as non-standard operating modes arising from strong external vibrations or electromagnetic interference (e.g., due to lightning during a thunderstorm). There is an approach known as control object identification, which has been rapidly developing recently. It provides certain options that allow for the identification of the control object (i.e., constructing its model) in an operational mode without interrupting the object’s operation [20,21]. A new and promising approach currently being developed is the use of neural networks to generate the most accurate possible model of the control object [15,16,22,23]. At the same time, another approach, which involves the estimation of disturbances using inverse models, is of significant interest [24,25,26,27].

It is known that there are techniques to mitigate or eliminate the influence of disturbances in case these are measurable. However, in case the external disturbance cannot be measured directly, the general idea is to estimate the disturbance (or its influences) indirectly using available measurable data and then apply known methods to compensate for the disturbance’s influences. The idea of indirect disturbance measurement naturally extends to the cases where uncertainties or the influences of uncertainties or inaccuracies in modeling system dynamics can be considered as disturbance components, as first presented in papers [28,29]. In this way, the influences of uncertainties were suppressed, and the stability of the system was enhanced. A group of such control algorithms can be distinguished, including unmodeled dynamics and parameter disturbances [30,31,32]. In the review by Chen et al. [26], this group of methods is referred to as disturbance/uncertainty estimation and attenuation (DUEA). A promising approach to constructing DUEA is the application of so-called inverse models.

Overall, the idea of using inverse models in automatic control systems is very simple, intuitive, and not new. However, this area is relatively under-researched and is associated with various interpretations and misunderstandings in terminology, and it is not standardized [26]. In this field, heuristic methods are often applied, and there are relatively few works with theoretical justifications for the problem. Below, there is a “naive” description of the basic ideas of using inverse models for control.

Let us consider the control object as a signal converter, with its input denoted as u(t), and output as y(t). Gu=y, where G is an operator acting on the input signal. Let $G^{-1}$ be the operator inverse to G, such that $GG^{-1}=I$, where I is the identity operator. Accordingly, $G{(G}^{-1}y)=y$. If we present the inverse model of the control object as a physical entity, such as a circuit board converting an electrical signal, or an electromechanical system, we can functionally define its operation as follows: in case the output of the control object is the input of the inverse model, then the output of the inverse model will match the signal that was applied to the input of the control object. Several notes should be made immediately:

Note 1: The inverse model can be physically unrealizable. For example, consider our control object as a delay line. In this case, naturally, we cannot construct a device capable of predicting the future. Although this is a rather crude example, in filter design theory, the established term “physically unrealizable filter” is essentially related to such limitations.

Note 2: In mathematics, there is the concept of “ill-posed problems according to Hadamard” [33]. It is well known that inverse problems often belong to this class. As an example, consider the case of a linear transformation y=Ax, where A is a matrix. If the determinant of matrix A is different from 0, then the inverse matrix $A^{-1}$ is defined, along with the corresponding inverse transformation ${x=A}^{-1}$. However, if matrix A is “ill-conditioned” (i.e., its determinant is close to zero), small changes in vector y during the inverse transformation can lead to large changes in the result of the inverse transformation x. This effect is well known for many practically important tasks, such as the numerical inversion of the Laplace transform, many problems in optics, and in applied mathematics as the solution for certain types of integral equations. Therefore, the problem of solving ill-posed problems (according to Hadamard) is a subject of intensive research in the field of applied mathematics, and currently, so-called “regularization methods” are used for this purpose [34,35]. In any case, “solving” an ill-posed problem involves restricting the domain in which one can seek the solution. Constructing an inverse model of the control object may lead to this kind of “ill-posed problem”.

**Figure 1:**
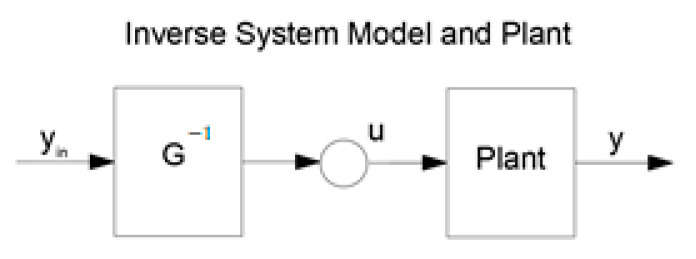
Inverse system model based on feedforward control system.

The diagram of the main “naive” idea of using an inverse model for control lies in creating a feedforward control system is shown in Figure 1.

**Figure 2:**
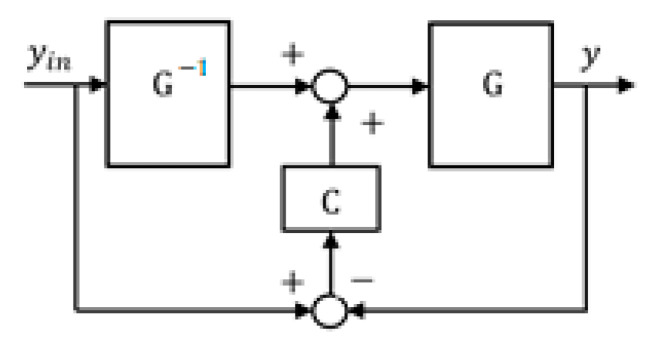
Inverse system model based on feedback control system.

In the “ideal” case, that is, in the absence of disturbances, and if the control system model accurately describes the control object, the system output will precisely follow the reference signal. However, in practice, such a “naive” scheme is not applicable. The next step in developing this approach is the introduction of feedback C into the control system, as schematically shown in Figure 2.

Thus, unlike the classical feedback control system design, which also mitigates the influence of uncertainties and unmeasurable disturbances, DUEA can be presented as a simple linear system that includes the real physical plant and its nominal model used for the controller design, and a stable filter, as detailed in the review [26]. DUEA methods provide a promising approach to overcoming the inherent structural limitations of feedback, offering advantages in tracking and disturbance rejection, as well as nominal control system performance. Although numerous applications have demonstrated the potential of DUEA methods [26], further research is needed to understand the drawbacks (or limitations) of these methods and to fully leverage their benefits.

This paper proposes a new control algorithm based on the inverse model of the control object, belonging to the group of DUEA methods. As an application of the general DUEA method, we consider a control system for a wheeled mobile robot with steering control. With the significant differences from the known inverse model-based control methods, the proposed new control idea involves the introduction of so-called reference filters, i.e., signal converters with adjustable parameters, and leads to a change in the given concept of the “inverse model”. Within the traditional inverse model-based control methods, the inverse model itself is rigidly determined by the control object. In the proposed method, the inverse model of the object is not used directly, but the disturbance compensation system is adjustable to ensure the desirable operating characteristics of the controller.

## 2. Research Motivation and Background

We consider the case where the control object is described by a mathematical model and Equation (1) describes the system dynamics:

(1) $${\dot{x}}_{0}=f\left(x_{0},u\right),$$

where $x_{0}$ is the state vector of the control object, $u\left(t\right)$ is the control signal vector, and f is the given function.

The output of the control object (the vector of quantities we control by applying the control signal $u\left(t\right)$ to the input) is denoted as$\;y\left(t\right)$. The vector $y\left(t\right)$ is related to the state vector $x_{0}\left(t\right)$ by the functional dependence in Equation (2):

(2) $$y\left(t\right)=g\left(x_{0}\left(t\right)\right).$$

We assume that the state vector of the control object is an observable quantity. The problem is that the disturbance of the state vector is not directly observable and requires indirect estimation methods to compensate for its influence. Below, we outline the main ideas underlying the proposed method.

For any fixed moment in time $t_{0}$, we can (formally) consider the system in Equation (3) as a system of algebraic equations for the unknown vectors $x_{0}^{*}\left(t_{0}\right)$ and $u^{*}\left(t_{0}\right)$, assuming that the vectors$\;{\dot{x}}_{0}\left(t_{0}\right)$ and $y\left(t_{0}\right)$ are known (given constants).

(3) $$\left\{\begin{array}{c} {\dot{x}}_{0}\left(t_{0}\right)=f\left(x_{0}^{*}\left(t_{0}\right),u^{*}\left(t_{0}\right)\right)\\ y\left(t_{0}\right)=g\left(x_{0}^{*}\left(t_{0}\right)\right) \end{array}\right.,$$

We assume that the solutions $x_{0}^{*}\left(t_{0}\right)$ and $u^{*}\left(t_{0}\right)$ in the form of Equation (4), where ${Inv}_{x}$ and ${Inv}_{u}$ are given functions, exist for any $t_{0}\in R$.

(4) $$x_{0}^{*}\left(t_{0}\right)={Inv}_{y}\left({\dot{x}}_{0}\left(t_{0}\right),y\left(t_{0}\right)\right)\;\;u^{*}\left(t_{0}\right)={Inv}_{u}\left({\dot{x}}_{0}\left(t_{0}\right),y\left(t_{0}\right)\right).$$

**Figure 3:**
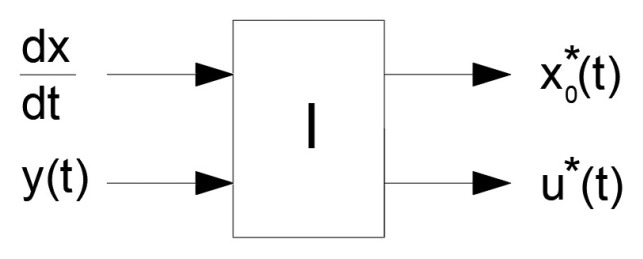
Inverse model of the control object as a signal converter.

Then, a signal converter, I, is formally defined, which we will refer to as the inverse model of the control object, with two inputs and two outputs, symbolically presented in Figure 3.

We emphasize that such a definition of the inverse model of the control object is unconventional.

Let us suppose we have successfully physically implemented such a signal converter. Now, let us assume that we have applied the signal ${\dot{x}}_{0}\left(t\right)$ to the first input of the converter, and the output of the actual control object $y\left(t\right)$ to the second input. It can be asserted that, in the presence of disturbances, the output $u^{*}\left(t\right)$ will not coincide with the control signal, which is expressed by Equation (5). Similarly, the other output of the inverse model $x_{0}^{*}\left(t\right)$ will not coincide with the signal $x_{0}\left(t\right)$.

(5) $$u^{*}\left(t\right)=u\left(t\right)+∆u\left(t\right).$$

“The inverse model” of the control object, as described above, is challenging to apply directly for the creation of a supervisory controller or for estimating the disturbance of the object’s state vector. The unique feature of the described method is that we introduce a new additional entity—a signal converter, which we call “reference filter”—to enable the indirect use of the inverse model in the supervisory controller and to quantitatively assess the disturbance of the system’s state vector. This signal converter is mathematically described in the same way as the control object. The current state of this object is described by the state vector $x\left(t\right)$ within the same state space as the control object’s state vector $x_{0}\left(t\right)$. The state vector of the signal converter $x\left(t\right)$ will model the “disturbance-free” state vector of the control object $x_{0}\left(t\right).\;$The time evolution of the state vector of the signal converter $x\left(t\right)$ is described by the dynamic equation of the object in Equation (6), which is of the same type as the dynamic equation of the control object in Equation (1).

(6) $$\dot{x}=h\left(x,y_{in}\right).$$

In Equation (6), $y_{in}\left(t\right)$ is the “control signal”—a signal applied externally to the input of the signal converter (as we will see later, the signal $y_{in}\left(t\right)$ can be interpreted as the setpoint signal input to the supervisor controller). The output $y_{out}\left(t\right)$ of our signal converter is a vector within the same state space as the output of the control object $y\left(t\right)$. The vector $y_{out}\left(t\right)$ is determined by the state vector of the signal converter $x\left(t\right)$ according to Equation (7). Equation (7) has the same form as Equation (2) in the description of the control object model:

(7) $$y_{out}\left(t\right)=\psi \left(x\left(t\right)\right).$$

The functions h and ψ in Equations (6) and (7) are calculated to satisfy two conditions:

**Condition** **1.** *In the static mode, the output of the converter $y_{out}$ will be set to the same constant value, C, that is applied to the input of the converter $y_{in}$, i.e., $y_{out}\left(t\right)=C=y_{in}(t)$. This condition, which can be referred to as the requirement for unity gain in the static mode, can be formulated algebraically as a condition imposed on the functions h and ψ. Specifically, if for some values of the variables x, y the equality $h\left(x,y\right)=0$ holds, then for these same values, the equality $\psi \left(x\right)=y$ must also hold.*

**Figure 4:**
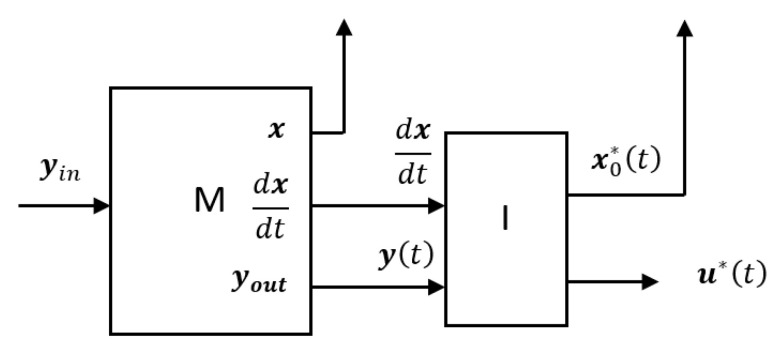
Series connection of the signal converter (“reference filter”) and the inverse model of the control object.

**Condition** **2.** If we apply the outputs $y_{out}\left(t\right)$ and $\dot{x}\left(t\right)$ of our signal converter M (“reference filter”) to the inputs $y\left(t\right)$ and $\frac{dx}{dt}$ of the inverse model of the control object, as shown in Figure 4, then no matter what signal $y_{in}(t)$ we apply to the input of our signal converter, at any time $t_{0}$, the state vector of the signal converter $x\left(t_{0}\right)$ will coincide with the output$\;x_{0}^{*}\left(t_{0}\right)$ of the inverse model of the control object, i.e., $\forall t\;x\left(t\right)=x_{0}^{*}\left(t\right)$ holds.

Since we are focusing on describing the main ideas of the method here, we do not provide the formulation of the algebraic conditions for the functions h and ψ, corresponding to Condition 2. These conditions will be formulated later for a specific type of system.

It is crucial to note that if our signal converter meets Conditions 1 and 2, then the series connection of the converter M and the inverse model of the control object, which we have just considered, can be regarded as a single signal converter block. This block transforms the input signal $y_{in}\left(t\right)$ into the output signal $u^{*}\left(t\right)$ (Figure 4). Such a block will represent the inverse model of the control object in the classical sense of the term.

This paper presents a robot control algorithm that includes a technique for compensating the dynamic changes and external influences, first described by Shadrin in the article [36] in relation to a number of synthetic and real objects; however, the classification and profound theoretical analysis of the method is presented here for the first time. This technique offers a number of advantages, including application to nonlinear and dynamic systems, intuitive presentation of input data, creation of multi-channel control using algebraic and analytical methods, precise control of object motion based on proven reference filters, and absence of static control errors. Currently, the use of intelligent control systems is considered popular and progressive for these purposes, such as when a neural network is used to compensate for model inaccuracies [15]. The interest in this topic is quite justified, but the application of such methods requires network training, which in turn requires special equipment, such as local positioning systems for trajectory control [15,16]. Thus, the use of neural networks necessitates a large number of experiments, special equipment, and time, whereas the proposed method of compensating for model inaccuracies and the uncertainties of random disturbances is adaptive in nature, and the compensation occurs automatically.

The main concept of the method is based on compensation of external additional influences on the state and output parameters of the system through an inverse model of the object. Unobserved impacts are determined through discrepancies between the real and the model values of the variables, which leads to the creation of feedback in the system, which was not originally intended but arose as a result of compensation for external noises. This method is adapted to work with linear systems having fixed parameters [36,37] and with a class of nonlinear dynamic systems presented in the form of State Dependent Coefficient (SDC) [38] and is used for the first time to control a four-wheeled mobile robot. The complexity of this analytical method is directly related to the complexity of the mathematical model of the control object, and the developed algorithms are characterized by robustness. Thus, the initial mathematical model of the robot should effectively implement the compensation method. When using this method, the number of controlled variables must correspond to the number of control actions. Solving general robot control problems is simplified by decomposing them into simpler subtasks. This decomposition, based on physical principles, has been implemented in a number of test cases [39,40] and includes automatic stabilization of the set speed and direction of the robot’s movement through adjustment of control signals to the motors. Direction is understood as the angle between the robot’s velocity vector at the target point and the positive direction of the *x*-axis of the stationary coordinate system. Stabilization of speed and direction plays a key role in controlling the robot, as it allows separate control channels and significantly simplifies the main task, moving the robot along a given trajectory. This is especially important for mobile robots that require precise positioning and navigation. Consistent speed provides smooth and predictable movement, which is critical for complex maneuvers or tasks that require precise trajectory tracking. Orientational stabilization helps the robot maintain a given course, which is important for accurate positioning in space. Stabilization systems can also quickly compensate for major disturbances, such as changes in load, robot mass, road unevenness, fluctuations in motor supply voltage, and others. These increase the stability and reliability of the robot under various conditions, which is of particular importance in industrial and operational environments. Stabilizing speed and direction does not only alleviate the robot’s control, but also increases its reliability, stability, and operational efficiency.

Moreover, recent trends in robotics involve additional application-level requirements as well, such as sustainability and ESG aspects. There already exists a standard [41] to follow for objective, ethically aligned robot design [42], and the UN’s Sustainable Development Goals have also been mapped to mobile robots particularly [43].

## 3. Problem Statement

We consider the control of a four-wheeled automotive-type robot (hereinafter referred to as the robot), which has two front wheels that are rotary and two rear wheels that are driven. DC electric motors are used to drive the swivel and drive the wheels. The middle of the rear axle of the driven wheels is taken as the target point. When compiling a mathematical model, we neglect the elasticity of all elements of the robot and assume that the wheels are in point contact with the surface and move along this surface without slipping or lateral displacement. Every mechanical system, driven by its own engine, has mass and therefore has inertia. However, the mass of the rear motor includes the mass of the robot itself, while the front motor drives only the wheels and the front mass is significantly less. Typically, electrical transients in drive motors settle much faster than mechanical transients. Thus, we do not consider the influence of the inertia of the front mass and electrical processes in the engines on the dynamic characteristics of the robot.

As mentioned above, the considered method for synthesizing the supervisor controller is suitable for control objects described by a mathematical model in the form of SDC. It is customary to distinguish between kinematic and dynamic models of wheeled robots. For some known and sufficiently accurate models, such as the one described in paper [44], the model can be presented in the form of SDC. However, such a model involves many state variables and parameters, such as moments of inertia, distances between axes, electrical parameters of drives, etc. This relative complexity of the model could be justified in our case when we were exploring issues such as the trajectory control of a mobile robot. However, since we were focusing on low-level control aspects, we preferred to use a simplified model with a steering wheel, allowing us to use the course angle and the modulus of the robot’s center of mass velocity vector as observable system parameters without separating the kinematic and dynamic models. Thus, we would like to emphasize that the new control method is certainly applicable to more realistic models of wheeled robots presented in the form of SDC. The choice of model is dictated solely by the clarity of interpreting the simulation results. Therefore, we use a simplified dynamic model of a four-wheeled mobile robot with steering, and holonomic constraints are not explicitly formulated, as will be shown later.

**Figure 5:**
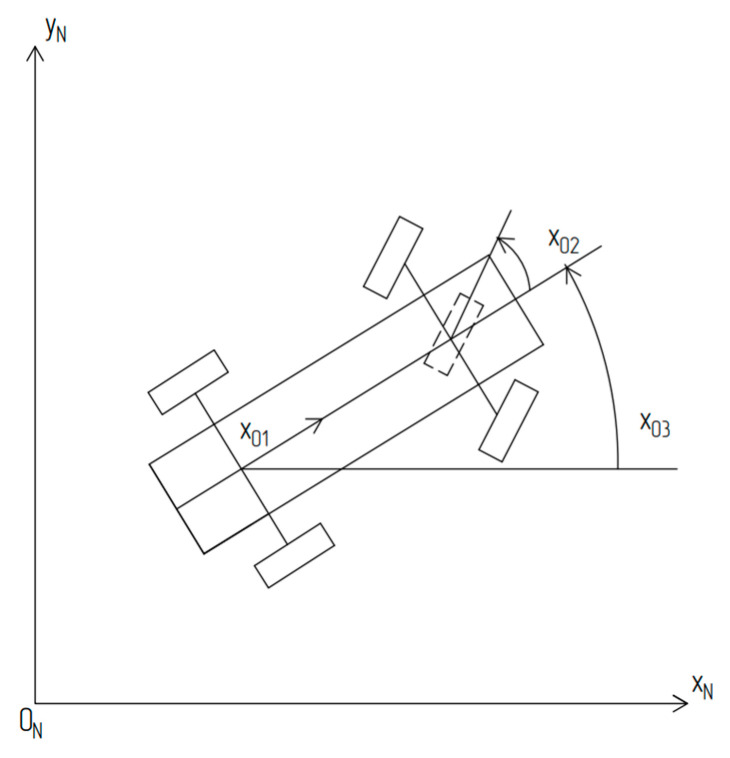
The diagram of the robot’s location on a plane in fixed coordinates $x_{N}$, $y_{N}$. $x_{01}$—robot speed; $x_{02}$—front wheel steering angle; $x_{03}$—robot course.

The diagram of the robot on the plane in fixed coordinates $x_{N}$, $y_{N}$ is shown in Figure 5.

Then, we can write Equation (8):

(8) $$\begin{array}{c} {\dot{x}}_{01}={-k}_{1}x_{01}+k_{2}u_{1}\\ {\dot{x}}_{02}=k_{3}u_{2} \end{array}$$

where $x_{01}$—robot linear speed module; $x_{02}$—angle of rotation of the front wheels relative to the longitudinal axis of the robot; $u_{1}$—rear drive motor voltage; $u_{2}$—front swing motor voltage; and $k_{1}$, $k_{2}$, and $k_{3}$—known coefficients depending on the electrical and mechanical parameters of the robot and the motors.

The acceleration of the center of mass ${\dot{x}}_{01}$ is inversely proportional to the linear speed and proportional to the voltage applied to the traction motor, while the angular speed of rotation of steering wheel ${\dot{x}}_{02}$ is proportional to the voltage applied to the steering actuator. We introduce a third state variable, $x_{03}$, which represents the angle between the direction of the velocity vector of the robot’s center of mass, v, and the $x_{N}$ axis of the fixed coordinate system. In the absence of wheel slip, the direction of vector v will align with the longitudinal symmetrical *y*-axis of the robot (see Figure 5). As will be shown below, the angular velocity of the robot ${\dot{x}}_{03}$ will be related to the state variables $x_{01}$ and $x_{02}$ by the kinematic constraint equation.

Suppose the trajectory of the wheeled robot is described by a smooth curve γ. γ is parameterized by the natural parameter s, and $s\left(t\right)=\int_{0}^{t}v\left(\tau \right)d\tau$, where $v=\left|v\right|$ the magnitude of the velocity vector $v\left(t\right)$ of the robot’s center of mass. That is, for any arbitrary moment in time $t_{0}>0$, the value of the parameter $s\left(t_{0}\right)$ corresponds to the distance traveled by the point (center of mass) along the trajectory described by the curve γ. It is evident that with this definition of the parameter s, the equality $\frac{ds}{dt}=v$ holds. The velocity vector of the robot’s center of mass v can be presented as in Equation (9):

(9) $$v\left(t\right)=v\left(t\right)\dot{\gamma }\left(s\left(t\right)\right)$$

where $\dot{\gamma }$ is the unit tangent vector to the curve γ. Differentiating Equation (9) with respect to time, we obtain Equation (10):

(10) $$\dot{v}\left(t\right)=\left(\frac{dv}{dt}\right)\dot{\gamma }\left(s\left(t\right)\right)+v\left(t\right)\left(\frac{ds}{dt}\right)\ddot{\gamma }\left(s\left(t\right)\right)$$

It is well known that the vector $\ddot{\gamma }\left(s\right)$ is orthogonal to the tangent vector $\dot{\gamma }\left(s\right)$ at any point of the curve γ. The magnitude of the vector $\ddot{\gamma }\left(s\right)$ is the curvature $k\left(s\right)$, i.e., $\left|\ddot{\gamma }\left(s\right)\;\right|=k\left(s\right)$. The curvature $k\left(s\right)$ is related to the radius $R\left(s\right)$ of the circle that is tangent to the curve γ at the point $\gamma \left(s\right)$ by Formula (11):

(11) $$k\left(s\right)=\frac{1}{R\left(s\right)}$$

Formula (10) can be written in the form of Equation (12):

(12) $$a=a_{tg}T+a_{n}N$$

where a is the acceleration vector of a point moving along the curve $\gamma ,\;a_{tg}=\frac{dv}{dt}$ is the tangential acceleration of the point, $a_{n}=\frac{v^{2}}{R}$ is the normal acceleration of the point, $T=\dot{\gamma }$ is the unit tangent vector, and $N=\frac{\ddot{y}}{\left|\ddot{\gamma }\;\right|}$ is the unit vector orthogonal to the T. Thus, Formula (10) can be interpreted as the well-known kinematic decomposition of the acceleration vector into tangential and normal components.

The curvature $k\left(s\right)$ can be interpreted as the limit in Equation (13):

(13) $$k\left(s\right)=\underset{∆s\to 0}{\lim}\frac{∠\left(\dot{\gamma }\left(s+∆s\right),\dot{\gamma }\left(s\right)\right)}{∆s},$$

Let us transition from the limit over the arc length interval of the curve to the limit over ∆t, a small time interval during which the point (the center of mass of the mobile robot) traverses the arc ∆s:

(14) $$k\left(s\right)=\frac{\underset{∆t\to 0}{\lim}\left(\frac{∠\left(\dot{\gamma }\left(s+∆s\right),\dot{\gamma }\left(s\right)\right)}{∆t}\right)}{\underset{∆t\to 0}{\lim}\frac{∆s}{∆t}}$$

It is obvious that

(15) $$\underset{∆t\to 0}{\lim}\frac{∆s}{∆t}=v$$

**Figure 6:**
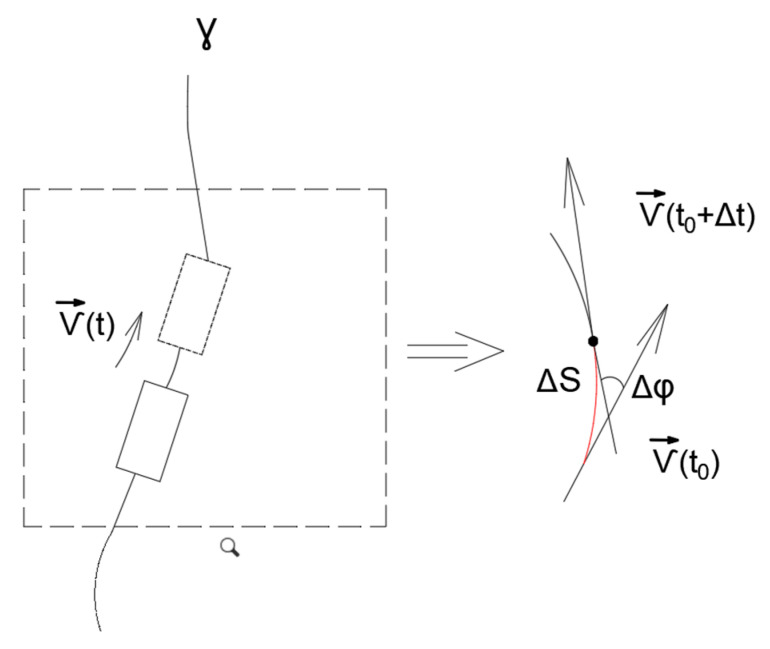
The connection of the curvature and the trajectory and the angular velocity of the mobile robot.

It is easy to observe that

(16) $$\underset{∆t\to 0}{\lim}\left(\frac{∠\left(\dot{\gamma }\left(s+∆s\right),\dot{\gamma }\left(s\right)\right)}{∆t}\right)=\underset{∆t\to 0}{\lim}\frac{∆\varphi }{∆t}=\omega$$

where $∆\varphi =∠\left(v\left(t+∆t\right),v\left(t\right)\right)$, as shown in Figure 6, and ω is an angular speed of the robot.

Taking into account Equations (15) and (16), Equation (14) takes the form of Equation (17):

(17) $$R\left(s\left(t\right)\right)\omega \left(t\right)=v\left(t\right)$$

**Figure 7:**
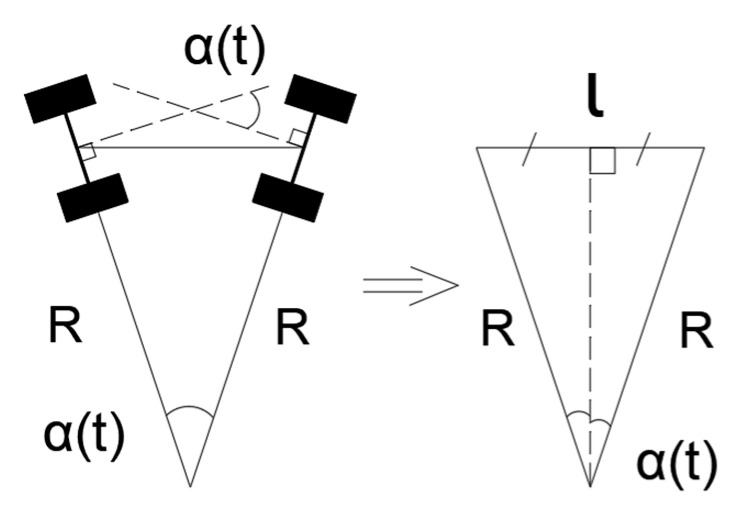
A schematic diagram of the steering wheel angle and the radius of the circle tangent to the trajectory.

If l is the distance between the front and the rear wheel axes of the robot, and α(t) is the current steering wheel angle, then as shown in Figure 7, the equality in Equation (18) holds true:

(18) $$\tan\left(\frac{\alpha \left(t\right)}{2}\right)=\frac{l}{2R\left(t\right)}$$

Considering Equations (17) and (18), we obtain Equation (19):

(19) $$\forall t\;\;\omega \left(t\right)-2\tan\left(\frac{\alpha \left(t\right)}{2}\right)\frac{v\left(t\right)}{l}=0.$$

Equation (19) defines a nonholonomic constraint for $\omega \left(t\right)$, $\alpha \left(t\right),$ and $v\left(t\right)$.

Assuming the angle α is small and considering that ${\omega =\dot{x}}_{03}$, Equation (19) can be rewritten in the form of Equation (20):

(20) $${\dot{x}}_{03}=k_{4}x_{01}\tan x_{02},$$

where $x_{03}$ is the course of the robot, $k_{4}=1/l$, and l is the distance between the rear and the front axles of the robot.

The steering angle of the front wheels is limited within reasonable limits by the double inequality in Equation (21):

(21) $$-x_{02max}\leq x_{02}\leq x_{02max}$$

The rotation speeds of the drive and rotary motors are also limited by double inequalities in Equation (22):

(22) $$\begin{array}{l} -{\dot{x}}_{01max}\leq {\dot{x}}_{01}\leq {\dot{x}}_{01max}\\ -{\dot{x}}_{02max}\leq {\dot{x}}_{02}\leq {\dot{x}}_{02max} \end{array}$$

In the expressions of Equations (21) and (22), $x_{02max}$, ${\dot{x}}_{01max}$, and ${\dot{x}}_{02max}$ are known constants.

**Note** **1.**  *According to (20), when the direction of movement of the robot ($x_{01})\;$changes sign and the angle of rotation of the front wheels ($x_{02}$) is the same, then the direction of course ($x_{03}$) also changes.*

The task is to develop an algorithm for controlling the speed and course of the robot using the equations of its mathematical model from Equations (1) and (20) by compensating for the dynamics of the object and impacts.

**Note** **2.** * If each wheel has its own motor, then there are still two control actions, but each of them, taking into account the curvature of the trajectory, is recalculated to its own pair of engines so that the wheels move without slipping. The recalculation problem is not considered in this article.*

## 4. Development of a Control Algorithm for a Four-Wheeled Robot

At the stage of analytical synthesis of the control algorithm, we assume that the inequalities in Equations (21) and (22) are fulfilled. The method for compensating the dynamics of an object and impacts includes the following steps [37,38]:

**Step** **1.** *Presentation of the mathematical model of the four-wheeled robot in the form of SDC. This presentation in general form in the absence of disturbances is written as follows in Equation (23):*

(23) $$\begin{array}{l} {\dot{x}}_{0}=A(x_{0})x_{0}+B(x_{0})u+f_{x},\\ y_{0}=C(x_{0})x_{0}+f_{y}, \end{array}$$

*where $x_{0}$—vector of state variables; $y_{0}—$vector of output variables; u—vector of control actions; $f_{x},\;f_{y}$—disturbance vectors corresponding to the state variables and output variables of the respective dimensions; and $A\left(x_{0}\right)$, $B\left(x_{0}\right)$, and $C(x_{0})$—matrices of corresponding dimensions. The block matrix is shown in Equation (24):*

(24) $$\left[\begin{array}{cc} A\left(x_{0}\right) & B\left(x_{0}\right)\\ C(x_{0}) & 0 \end{array}\right]$$

*This equation must be non-degenerate.*

Let us write initial Equations (8) and (20) of the mathematical model of the robot in state space as Equation (25):

(25) $$\begin{array}{l} {\dot{x}}_{01}=-k_{1}x_{01}+k_{2}u_{1}\\ {\dot{x}}_{02}=k_{3}u_{2}\\ {\dot{x}}_{03}=k_{4}x_{01}\tan x_{02}.\\ y_{01}=x_{01},\\ y_{02}=x_{03}. \end{array}$$

To present it as SDC, the right side of the third equation from Equation (25) is multiplied and divided with $x_{02}$; at the same time, to eliminate uncertainty, it must be ensured that $x_{02}\neq 0$. Then, Equation (25) in SDC form can be presented as Equation (26):

(26) $$\begin{array}{l} \left[\begin{array}{c} {\dot{x}}_{01}\\ {\dot{x}}_{02}\\ {\dot{x}}_{03} \end{array}\right]=\left[\begin{array}{ccc} -k_{1} & 0 & 0\\ 0 & 0 & 0\\ 0 & \left(k_{4}x_{01}\tan x_{02}\right)/x_{02} & 0 \end{array}\right]\left[\begin{array}{c} x_{01}\\ x_{02}\\ x_{03} \end{array}\right]+\left[\begin{array}{cc} k_{2} & 0\\ 0 & k_{3}\\ 0 & 0 \end{array}\right]\left[\begin{array}{c} u_{1}\\ u_{2} \end{array}\right],\\ \left[\begin{array}{c} y_{01}\\ y_{02} \end{array}\right]=\left[\begin{array}{ccc} 1 & 0 & 0\\ 0 & 0 & 1 \end{array}\right]\left[\begin{array}{c} x_{01}\\ x_{02}\\ x_{03} \end{array}\right],\;x_{02}\neq 0. \end{array}$$

From Equation (26), we have Equations (27) and (28):

(27) $$A(x_{0})=\left[\begin{array}{ccc} -k_{1} & 0 & 0\\ 0 & 0 & 0\\ 0 & \left(k_{4}x_{01}\tan x_{02}\right)/x_{02} & 0 \end{array}\right]$$

$$B\left(x_{0}\right)=\left[\begin{array}{cc} k_{2} & 0\\ 0 & k_{3}\\ 0 & 0 \end{array}\right]$$

(28) $$C\left(x_{0}\right)=\left[\begin{array}{ccc} 1 & 0 & 0\\ 0 & 0 & 1 \end{array}\right]$$

The block matrix in Equation (24), taking into account Equations (27) and (28), has the form seen in Equation (29):

(29) $$\left[\begin{array}{cc} A(x_{0}) & B(x_{0})\\ C(x_{0}) & 0 \end{array}\right]=\left[\begin{array}{ccccc} -k_{1} & 0 & 0 & k_{2} & 0\\ 0 & 0 & 0 & 0 & k_{3}\\ 0 & \left(k_{4}x_{01}\tan x_{02}\right)/x_{02} & 0 & 0 & 0\\ 1 & 0 & 0 & 0 & 0\\ 0 & 0 & 1 & 0 & 0 \end{array}\right]$$

**Step** **2.** *Obtaining inverse models of the control object using the task and disturbance channels. The inverse model (IM) for the channel of tasks for the speed and course of the robot is written in the form of Equation (30):*

(30) $$\begin{array}{l} x=E(x_{0})\dot{x}+F(x_{0}){y^{\prime }}_{\varphi },\\ u_{z}=G(x_{0})\dot{x}+H(x_{0}){y^{\prime }}_{\varphi }, \end{array}$$

*Using the channel for processing disturbances to state variables, we obtain Equation (31):*

(31) $$\begin{array}{l} \tilde{x}=E(x_{0})\dot{\tilde{x}}+F(x_{0})\Delta {y^{\prime }}_{\varphi },\\ \Delta u=G(x_{0})\dot{\tilde{x}}+H(x_{0})\Delta {y^{\prime }}_{\varphi },\;u=u_{z}+\Delta u, \end{array}$$

*where $x={\left[\begin{array}{ccc} x_{1} & x_{2} & x_{3} \end{array}\right]}^{T}$—vector of state variables in (30), corresponding to the vector $x_{0}$; $\tilde{x}={\left[\begin{array}{ccc} {\tilde{x}}_{1} & {\tilde{x}}_{2} & {\tilde{x}}_{3} \end{array}\right]}^{T}$—vector of state variables in (31); ${y^{\prime }}_{\varphi }={\left[\begin{array}{cc} {y^{\prime }}_{\varphi 1} & {y^{\prime }}_{\varphi 2} \end{array}\right]}^{T}$—vector of input variables in (31); $\Delta {y^{\prime }}_{\varphi }={\left[\begin{array}{cc} \Delta {y^{\prime }}_{\varphi 1} & \Delta {y^{\prime }}_{\varphi 2} \end{array}\right]}^{T}$—vector of input variables in (31); $u={\left[\begin{array}{cc} u_{1} & u_{2} \end{array}\right]}^{T}$—vector of control signals to the robot drive and rotary motors; and $E(x_{0})$, $F(x_{0})$, $G(x_{0})$, and $H(x_{0})$—matrices of corresponding dimensions. $\Delta {y^{\prime }}_{\varphi }$ will be defined further in Equation (36).*

**Note** **3.** * Inverse models are physically unrealizable and are intermediate constructions for the synthesis of a control algorithm.*

The matrices E(x_0_), F(x_0_), G(x_0_), and H(x_0_) in Equations (30) and (31) are the results of inverting the block matrix of Equation (29) as Equation (32):

(32) $$\left[\begin{array}{cc} E(x_{0}) & F(x_{0})\\ G(x_{0}) & H \end{array}\right]={\left[\begin{array}{cc} A(x_{0}) & B(x_{0})\\ C(x_{0}) & 0 \end{array}\right]}^{-1}$$

For a given robot, this is written as Equation (33):

(33) $$\left[\begin{array}{cc} E(x_{0}) & F(x_{0})\\ G(x_{0}) & H(x_{0}) \end{array}\right]={\left[\begin{array}{ccccc} -k_{1} & 0 & 0 & k_{2} & 0\\ 0 & 0 & 0 & 0 & k_{3}\\ 0 & \left(k_{4}x_{01}\tan x_{02}\right)/x_{02} & 0 & 0 & 0\\ 1 & 0 & 0 & 0 & 0\\ 0 & 0 & 1 & 0 & 0 \end{array}\right]}^{-1}$$

Thus, it is assumed that the original functional matrix for inversion in Equation (33) is non-degenerate (non-singular) within the range of its variables. A control object with such an original matrix is called output controllable [45]. Performing the inversion in Equation (33) using the analytical method (simple but cumbersome intermediate transformations are omitted here and below), we obtain Equations (34) and (35):

(34) $$E(x_{0})=\left[\begin{array}{ccc} 0 & 0 & 0\\ 0 & 0 & x_{02}/\left(k_{4}x_{01}\tan x_{02}\right)\\ 0 & 0 & 0 \end{array}\right]$$

(35) $$\begin{array}{l} F=\left[\begin{array}{cc} 1 & 0\\ 0 & 0\\ 0 & 1 \end{array}\right]\\ G=\left[\begin{array}{ccc} 1/k_{2} & 0 & 0\\ 0 & 1/k_{3} & 0 \end{array}\right]\\ H=\left[\begin{array}{cc} k_{1}/k_{2} & 0\\ 0 & 0 \end{array}\right] \end{array}$$

**Step** **3.** *Development of reference filters for a closed-loop system using task and disturbance channels. The filters set the dynamic and static characteristics of a closed-loop control system through the channels of assignments and processing of disturbances. The combined equations of reference filters for these channels have the following form in Equation (36):*

(36) $$\begin{array}{l} {\dot{x}}_{\varphi }=\Phi_{1}(x_{0})x_{\varphi }+\Phi_{2}(x_{0})y,\\ {\dot{\tilde{x}}}_{\varphi }=\Phi_{r1}(x_{0}){\tilde{x}}_{\varphi }+f_{x},\\ y_{0}=\Phi_{3}(x_{0})x_{\varphi }+\Phi_{r3}(x_{0}){\tilde{x}}_{\varphi },\\ \Delta {y^{\prime }}_{\varphi }=\Phi_{r3}(x_{0}){\tilde{x}}_{\varphi }, \end{array}$$

*where $x_{\varphi }={\left[\begin{array}{ccc} x_{\varphi 1} & x_{\varphi 2} & x_{\varphi 3} \end{array}\right]}^{T}$—vector of state variables of the reference filter along task channels; ${\tilde{x}}_{\varphi }={\left[\begin{array}{ccc} {\tilde{x}}_{\varphi 1} & {\tilde{x}}_{\varphi 2} & {\tilde{x}}_{\varphi 3} \end{array}\right]}^{T}={\left[\begin{array}{ccc} x_{01}-x_{\varphi 1} & x_{02}-x_{\varphi 2} & x_{03}-x_{\varphi 3} \end{array}\right]}^{T}$—vector of state variables of the reference filter along the channels for processing disturbances to state variables; $x_{01}$, $x_{92}$, and $x_{03}$—robot state variables; $y={\left[\begin{array}{cc} y_{1} & y_{2} \end{array}\right]}^{T}$—vector of tasks for speed and course; $y_{0}={\left[\begin{array}{cc} y_{01} & y_{02} \end{array}\right]}^{T}$—robot speed and heading vector; and $f_{\varphi }={\left[\begin{array}{ccc} f_{\varphi 1} & f_{\varphi 2} & f_{\varphi 3} \end{array}\right]}^{T}$—vector of disturbances on state variables*.

**Note** **4.** *Equation (36) represents intermediate constructions for the synthesis of a controller and does not appear explicitly in the controller*.

Matrices $\Phi_{1}\left(x_{0}\right),\Phi_{2}(x_{0})$, $\Phi_{3}(x_{0})$, $\Phi_{r1}(x_{0})$, and $\Phi_{r3}(x_{0})$ must fulfill functional Equations (37) and (38):

(37) $$\begin{array}{l} E(x_{0})\Phi_{1}(x_{0})+F\Phi_{3}(x_{0})=I,\\ E(x_{0})\Phi_{r1}(x_{0})+F\Phi_{r3}(x_{0})=I, \end{array}$$

(38) $$\Phi_{2}(x_{0})=-\Phi_{1}(x_{0})F$$

where I is the diagonal identity matrix. Equations (37) and (38) were first presented in article [45] for the synthesis of a control algorithm for a nonlinear object; the validity of these equations was confirmed by the simulation results. Equation (38) provides unit transmission coefficients of the reference filter of Equation (36) over control channels in static modes. Equation (37) is the condition for the physical implementation of a two-channel speed and course controller, built on the basis of inverse models in Equations (30) and (31). Equations (37) and (38) do not completely determine the matrices in Equation (36). The remaining degrees of freedom are used to set the dynamic properties of the closed-loop control system. It is not difficult to establish that Equation (37) provides 9+9+6=24 conditions for determining the elements of the matrices $\Phi_{1}(x_{0})$, $\Phi_{2}(x_{0})$, $\Phi_{3}(x_{0})$, $\Phi_{r1}(x_{0})$, and $\Phi_{r3}(x_{0})$ for a given task. At the same time, these matrices contain 9+9+6+6+6=36 unknown elements. Therefore, 36 • 24 = 12 matrix elements can be specified arbitrarily.

After a series of trials and errors, using the results of previous developments [33,34,35], we accept the elements of the first two rows of the matrices as arbitrarily specified $\Phi_{1}(x_{0})$ and $\Phi_{r1}(x_{0})$ (39) elements:

(39) $$\begin{array}{c} \left[\begin{array}{ccc} \varphi_{11} & \varphi_{12} & \varphi_{13}\\ \varphi_{21} & \varphi_{22} & \varphi_{23} \end{array}\right]\\ \left[\begin{array}{ccc} \varphi_{r11} & \varphi_{r12} & \varphi_{r13}\\ \varphi_{r21} & \varphi_{r22} & \varphi_{r23} \end{array}\right] \end{array}$$

These elements determine the dynamic characteristics of the reference filters and, consequently, the quality of operation of the closed-loop control system. According to their physical meanings, the coefficients $\varphi_{11}$, $\varphi_{22}$ and $\varphi_{r11}$, $\varphi_{r22}\;$in Equation (39) determine the speed of the system through the control and disturbance channels; these are negative (for system stability) numbers. Cross-connections between $x_{\varphi 1}$ (speed in the reference filter) and $x_{\varphi 2}$ (angle of rotation of the front wheels) are evaluated (cross-connections between ${\tilde{x}}_{\varphi 1}\;and\;{\tilde{x}}_{\varphi 2}$ are not important in this case, so the coefficients $\varphi_{12}$,$\;\varphi_{21}$ and $\;\varphi_{r12}$,$\;\varphi_{r21}$ must be equal to zero). To ensure stability and quality of control along the heading channel, at the robot’s forward movement from the heading coordinate, there must be negative feedback on the coordinates $x_{\varphi 3}$ and $x_{\varphi 2}$ (front wheel steering angle). That is, the coefficients $\varphi_{23}$ and $\varphi_{r23}$ when the robot moves forward should be less than zero. On the contrary, when moving backwards, these coefficients must be positive (see Note 1). Therefore, the sign of these coefficients should depend on the sign of the robot speed. It should be noted that to eliminate the change in the angle of rotation of the front wheels at $x_{01}=0$, there must be ${\mathrm{sgnx}}_{01}=0$. Then, the final elements of the first two rows of matrices are as presented in expression Equation (40):

(40) $$\begin{array}{c} \left[\begin{array}{ccc} -\varphi_{11} & 0 & 0\\ 0 & -\varphi_{22} & -\varphi_{23}{sgnx}_{01} \end{array}\right]\\ \left[\begin{array}{ccc} -\varphi_{r11} & 0 & 0\\ 0 & -\varphi_{r22} & -\varphi_{r23}{sgnx}_{01} \end{array}\right], \end{array}$$

To determine the matrix $\Phi_{3}(x_{0})$ and the remaining coefficients of the third row of the matrix $\Phi_{1}(x_{0})$, taking into account Equation (40), we write the 1st Equation (37) $E(x_{0})\Phi_{1}(x_{0})+F(x_{0})\Phi_{3}(x_{0})=1$ as Equation (41):

(41) $$\begin{array}{l} \left[\begin{array}{ccc} 0 & 0 & 0\\ 0 & 0 & x_{02}/\left(k_{4}x_{01}\tan x_{02}\right)\\ 0 & 0 & 0 \end{array}\right]\left[\begin{array}{ccc} -\varphi_{11} & 0 & 0\\ 0 & -\varphi_{22} & -\varphi_{23}{sgnx}_{01}\\ \varphi_{31} & \varphi_{32} & \varphi_{33} \end{array}\right]+\\ +\left[\begin{array}{cc} 1 & 0\\ 0 & 0\\ 0 & 1 \end{array}\right]\left[\begin{array}{ccc} \alpha_{11} & \alpha_{12} & \alpha_{13}\\ \alpha_{21} & \alpha_{22} & \alpha_{23} \end{array}\right]=\left[\begin{array}{ccc} 1 & 0 & 0\\ 0 & 1 & 0\\ 0 & 0 & 1 \end{array}\right], \end{array}$$

where $\varphi_{ij}$—matrix elements $\Phi_{1}(x_{0})$; and $\alpha_{ij}$—matrix elements $\Phi_{3}(x_{0})$. Performing matrix multiplication in Equation (41) and equating the corresponding matrix elements on the right and left sides of the equality in Equation (41), we obtain Equation (42):

(42) $$\Phi_{1}(x_{0})=\left[\begin{array}{ccc} -\varphi_{11} & 0 & 0\\ 0 & -\varphi_{22} & -\varphi_{23}{sgnx}_{01}\\ 0 & \left(k_{4}x_{01}{tanx}_{02}\right)/x_{02} & 0 \end{array}\right],$$

(43) $$\Phi_{2}=\left[\begin{array}{cc} \varphi_{11} & 0\\ 0 & \varphi_{23}{sgnx}_{01}\\ 0 & 0 \end{array}\right],\;\Phi_{2}=\left[\begin{array}{cc} \varphi_{11} & 0\\ 0 & \varphi_{23}{sgnx}_{01}\\ 0 & 0 \end{array}\right]$$

Carrying out similar actions for the second equation in Equation (37), we receive Equation (44):

(44) $$\begin{array}{l} \Phi_{r1}(x_{0})=\left[\begin{array}{ccc} -\varphi_{r11} & 0 & 0\\ 0 & -\varphi_{r22} & -\varphi_{r23}{sgnx}_{01}\\ 0 & \left(k_{4}x_{01}\tan x_{02}\right)/x_{02} & 0 \end{array}\right].\\ \Phi_{r3}=\left[\begin{array}{ccc} 1 & 0 & 0\\ 0 & 0 & 1 \end{array}\right] \end{array}$$

**Step** **4.** *Determining the control algorithm for the four-wheeled robot. According to the approach previously described in paper [38], the equations of the control algorithm can be presented as Equation (45):*

(45) $$\begin{array}{l} {\dot{x}}_{\varphi }=R_{1}(x_{0})x_{\varphi }+\Phi_{2}\varepsilon ,\;\\ u=R_{3}(x_{0})x_{\varphi }+N_{1}(x_{0}){\tilde{x}}_{0\varphi }+P(x_{0})\varepsilon , \end{array}$$

(46) $$\begin{array}{l} R_{1}(x_{0})=\Phi_{1}(x_{0})+\Phi_{2}\Phi_{3},\;R_{3}(x_{0})=N(x_{0})+P\Phi_{3},\;P=G\Phi_{2},\\ N(x_{0})=G\Phi_{1}(x_{0})+H\Phi_{3},\;N_{1}(x_{0})=G\Phi_{r1}(x_{0})+H\Phi_{3},\\ {\tilde{x}}_{0\varphi }=x_{0}-x_{\varphi },\;\;\varepsilon =y-y_{0}. \end{array}$$

**Figure 8:**
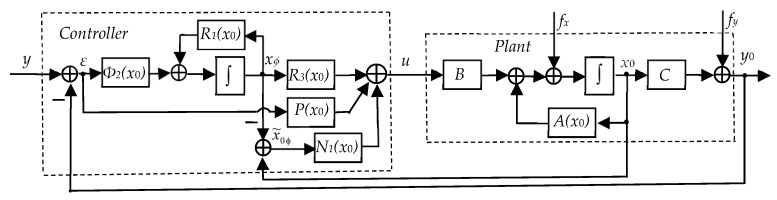
A block diagram of the robot’s speed and course control system.

*where $y={\left[\begin{array}{cc} y_{1} & y_{2} \end{array}\right]}^{T}$ is the vector of specified values of controlled variables ($y_{1}$—course assignment; $y_{2}$—course assignment). Figure 8 shows a block diagram of the robot’s speed and course control system*.

Using Equations (34) and (35) and Equations (42)–(44) we calculate the controller matrices in Equations (45) and (46) as Equations (47)–(51):

(47) $$R_{1}(x_{0})=\left[\begin{array}{ccc} 0 & 0 & 0\\ 0 & -\varphi_{22} & 0\\ 0 & \left(k_{4}x_{01}\tan x_{02}\right)/x_{02} & 0 \end{array}\right],$$

(48) $$N(x_{0})=\left[\begin{array}{ccc} \left(k_{1}-\varphi_{11}\right)/k_{2} & 0 & 0\\ 0 & -\varphi_{22}/k_{3} & -\left(\varphi_{23}{sgnx}_{01}\right)/k_{3} \end{array}\right].$$

(49) $$N_{1}(x_{0})=\left[\begin{array}{ccc} \left(k_{1}-\varphi_{r11}\right)/k_{2} & 0 & 0\\ 0 & -\varphi_{r22}/k_{3} & -\left(\varphi_{r23}{sgnx}_{01}\right)/k_{3} \end{array}\right],$$

$$N(x_{0})=\left[\begin{array}{ccc} \left(k_{1}-\varphi_{11}\right)/k_{2} & 0 & 0\\ 0 & -\varphi_{22}/k_{3} & -\left(\varphi_{23}{sgnx}_{01}\right)/k_{3} \end{array}\right]$$

(50) $$R_{3}(x_{0})=\left[\begin{array}{ccc} k_{1}/k_{2} & 0 & 0\\ 0 & -\varphi_{22}/k_{3} & 0 \end{array}\right],$$

(51) $$P(x_{0})=\left[\begin{array}{cc} \varphi_{11}/k_{2} & 0\\ 0 & \left(\varphi_{23}{sgnx}_{01}\right)/k_{3} \end{array}\right],$$

We substitute Equations (47)–(51) into Equation (45) and obtain Equation (52):

(52) $$\begin{array}{l} \left[\begin{array}{c} {\dot{x}}_{\varphi 1}\\ {\dot{x}}_{\varphi 2}\\ {\dot{x}}_{\varphi 3} \end{array}\right]=\left[\begin{array}{ccc} 0 & 0 & 0\\ 0 & -\varphi_{22} & 0\\ 0 & \left(k_{4}x_{01}\tan x_{02}\right)/x_{02} & 0 \end{array}\right]\left[\begin{array}{c} x_{\varphi 1}\\ x_{\varphi 2}\\ x_{\varphi 3} \end{array}\right]+\left[\begin{array}{cc} \varphi_{11} & 0\\ 0 & \varphi_{23}{sgnx}_{01}\\ 0 & 0 \end{array}\right]\left[\begin{array}{c} \varepsilon_{1}\\ \varepsilon_{2} \end{array}\right],\\ \left[\begin{array}{c} u_{1}\\ u_{2} \end{array}\right]=\left[\begin{array}{ccc} k_{1}/k_{2} & 0 & 0\\ 0 & -\varphi_{22}/k_{3} & 0 \end{array}\right]\left[\begin{array}{c} x_{\varphi 1}\\ x_{\varphi 2}\\ x_{\varphi 3} \end{array}\right]+\\ +\left[\begin{array}{ccc} \left(k_{1}-\varphi_{r11}\right)/k_{2} & 0 & 0\\ 0 & -\varphi_{r22}/k_{3} & -\left(\varphi_{r23}{sgnx}_{01}\right)/k_{3} \end{array}\right]\left[\begin{array}{c} {\tilde{x}}_{\varphi 1}\\ {\tilde{x}}_{\varphi 2}\\ {\tilde{x}}_{\varphi 3} \end{array}\right]+\left[\begin{array}{cc} \varphi_{11}/k_{2} & 0\\ 0 & \left(\varphi_{23}{sgnx}_{01}\right)/k_{3} \end{array}\right]\left[\begin{array}{c} \varepsilon_{1}\\ \varepsilon_{2} \end{array}\right],\\ {\tilde{x}}_{\varphi }={\left[\begin{array}{ccc} {\tilde{x}}_{\varphi 1} & {\tilde{x}}_{\varphi 2} & {\tilde{x}}_{\varphi 3} \end{array}\right]}^{T}={\left[\begin{array}{ccc} x_{01}-x_{\varphi 1} & x_{02}-x_{\varphi 2} & x_{03}-x_{\varphi 3} \end{array}\right]}^{T}. \end{array}$$

From Equation (52), we obtain the control algorithm in scalar form as Equation (53):

(53) $$\begin{array}{l} {\dot{x}}_{\varphi 1}=\varphi_{11}\varepsilon_{1},\\ {\dot{x}}_{\varphi 2}=-\varphi_{22}x_{\varphi 2}+\varphi_{23}\varepsilon_{2}{\mathrm{sgnx}}_{01},\\ {\dot{x}}_{\varphi 3}=\left(\left(k_{4}x_{01}{tanx}_{02}\right)/x_{02}\right)x_{\varphi 2},\\ u_{1}=\left(k_{1}/k_{2}\right)x_{\varphi 1}+\left(\left(k_{1}-\varphi_{r11}\right)/k_{2}\right){\tilde{x}}_{\varphi 1}+\left(\varphi_{11}/k_{2}\right)\varepsilon_{1},\\ u_{2}=-\left(\varphi_{22}/k_{3}\right)x_{\varphi 2}-\left(\varphi_{r22}/k_{3}\right){\tilde{x}}_{\varphi 2}-\left(\left(\varphi_{r23}{sgnx}_{01}\right)/k_{3}\right){\tilde{x}}_{\varphi 3}+\left(\left(\varphi_{23}{sgnx}_{01}\right)/k_{3}\right)\varepsilon_{2},\\ {\tilde{x}}_{\varphi 1}=x_{01}-x_{\varphi 1};\;{\tilde{x}}_{\varphi 2}=x_{02}-x_{\varphi 2};\;{\tilde{x}}_{\varphi 3}=x_{03}-x_{\varphi 3};\\ \varepsilon_{1}=y_{1}-y_{01};\;\varepsilon_{2}=y_{2}-y_{02}. \end{array}$$

## 5. Analysis of the Suggested Control System

We analyzed the control system of a four-wheeled robot using computer modeling. The modeling scheme included a mathematical model, Equation (25), and a controller, Equation (53), combined into a single system. When modeling, we had to take numerical values of the parameters of a four-wheeled robot and select free coefficients of reference filters. For simplicity, all coefficients of the $k_{1}$ and $k_{4}$ robot models were taken as equal to one. The freely specified coefficients of the reference filter in Equation (40) were also taken to be equal to unity, with the exception of $\phi_{22}$ and $\phi_{r22}$ which were equal to 1.73. Such values ensured an aperiodic transient process in a second-order dynamic system along the channel: a control signal to the front wheel turning motor—the robot’s course and moderate amplitudes of control actions. The controller model included a block that ensured the fulfillment of the condition $x_{02}\neq 0$. This block implemented the function in Equation (54):

(54) $$x_{0}=\left\{\begin{array}{c} 0.001\;\\ x_{0} \end{array}\right.\begin{array}{c} at\;\left|x_{0}\right|\leq 0.001\\ at\;\left|x_{0}\right|>0.001 \end{array},$$

In addition, to fulfill the inequality in Equation (21) in the plant and controller models, integrators with saturation were used. The saturation levels in the simulation were assumed to be ±0.8 radians, which corresponded to the maximum steering angle of the front wheels of ±45.8 degrees.

**Figure 9:**
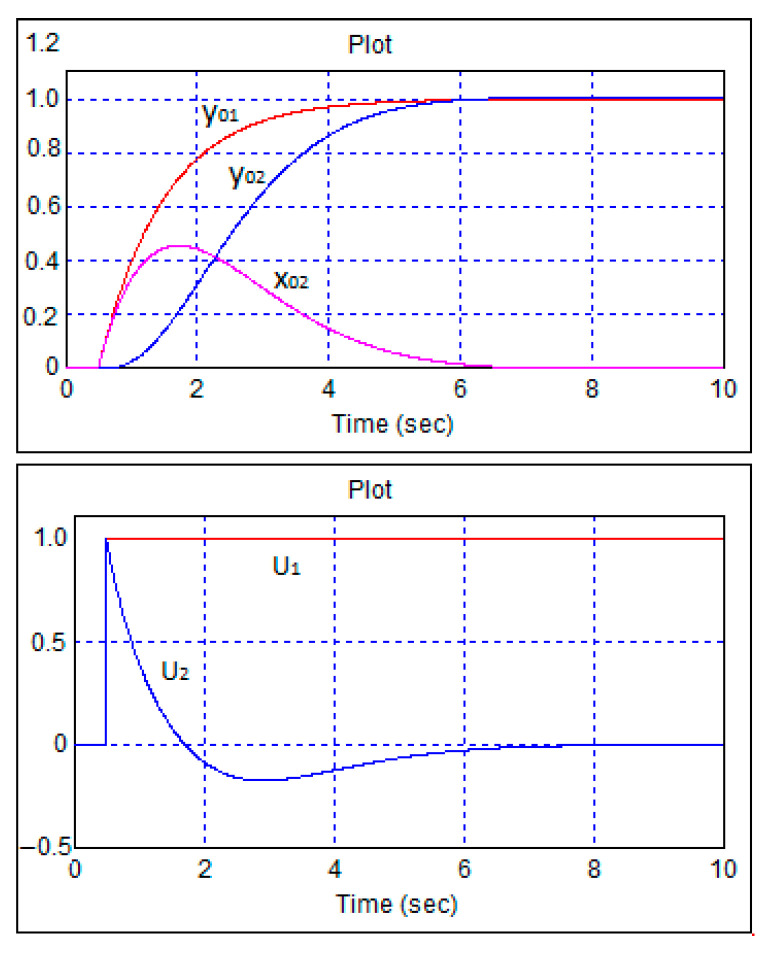
Transient processes in the robot control system during single-step changes in speed and heading tasks and forward movement. The designations of the variables correspond to their designations in Equations (25) and (52).

Figure 9 shows transient processes in the robot control system with single-step changes in speed and heading tasks and forward movement. For clarity, here and below, the steps were fed with a delay of 0.5 s.

**Figure 10:**
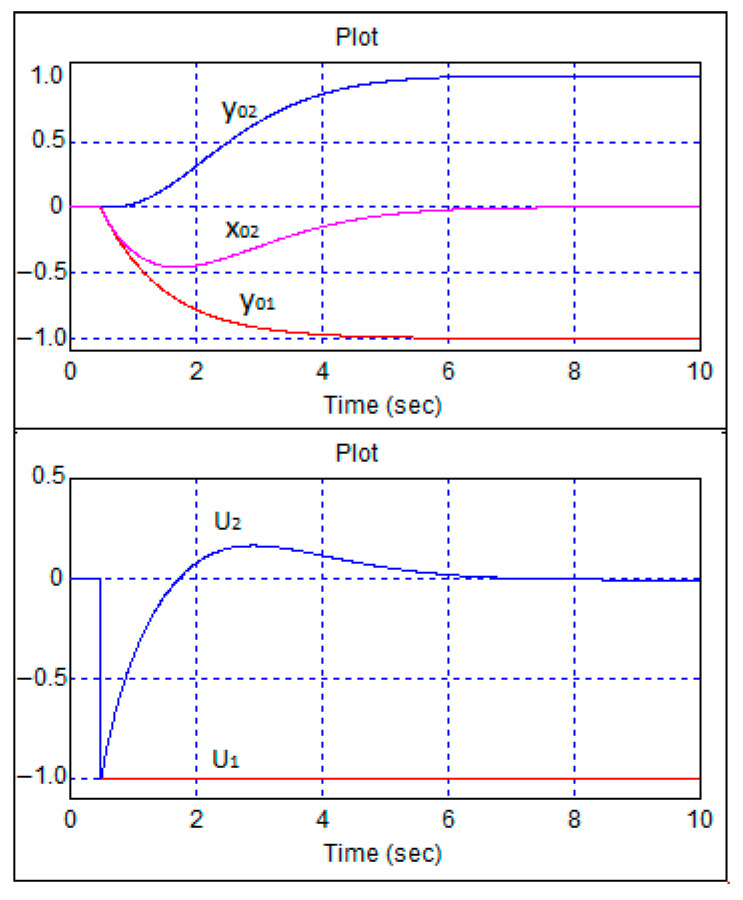
Transient processes in the robot control system during single-step changes in speed and heading tasks and backward movement.

As can be seen from Figure 9, aperiodic transient processes occurred. Figure 10 shows the same transient processes when moving backwards.

**Figure 11:**
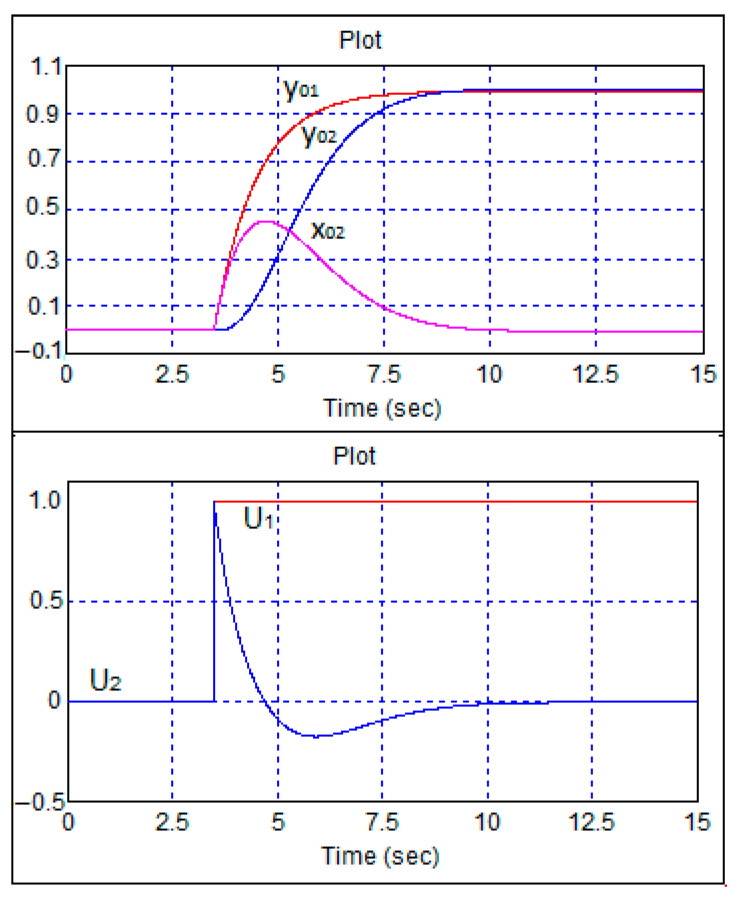
Transient processes in the robot control system during single-step changes in speed and heading tasks. The speed command changes 3 s after the heading command was changed.

As can be seen from Figure 10, in the case of reverse motion, aperiodic transient processes were also observed. Figure 11 shows transient processes when the step change in the speed task was delayed in relation to the heading task by 3 s.

As can be seen in Figure 11, when the heading task and zero speed appeared, the front wheels did not move. Further, after the movement started, the robot’s course came into compliance with the task, after which the wheels were set to the zero position. This corresponded to the physics of the robot control process.

**Figure 12:**
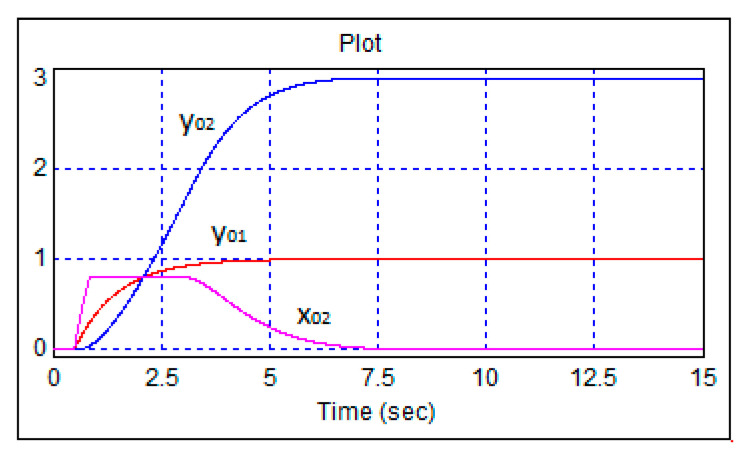
Transient processes in the robot control system with a single-step change in the speed task and 3 radians per course.

To study the effect of restrictions on the rotation of the front wheels, the stepwise heading task was increased to three radians at a unit speed. The transition functions can be seen in Figure 12.

In accordance with Figure 12, the front wheels of the robot turned to a stop equal to 0.8 radians, but this only delayed the transient processes and did not affect the quality of control.

To illustrate the movement of the robot in fixed coordinates with automatic stabilization of speed and course, the actual speed and course were calculated by solving the differential in Equation (55) on the model:

(55) $$\begin{array}{c} \dot{x_{N}}=x_{01}\cos x_{03},\\ \dot{y_{N}}=x_{01}\sin x_{03,} \end{array}$$

where$\;x_{N}$ and $y_{N}$ are robot coordinates in fixed coordinates.

**Figure 13:**
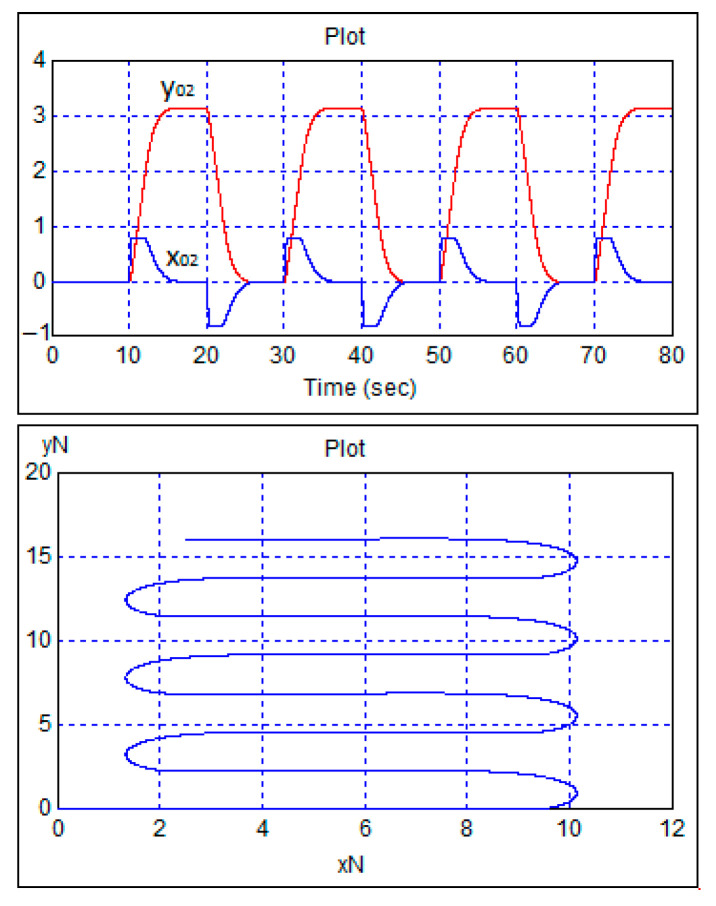
The robot control signals presented (**top figure**) in the case when the movement of the robot was in fixed coordinates (**bottom figure**) and when the course assignment changed by ±180 degrees every 10 s.

Figure 13 shows the programmed movement of the robot in fixed coordinates at a speed of 1 m/s and when changing the course assignment by ±180 degrees every 10 s.

**Figure 14:**
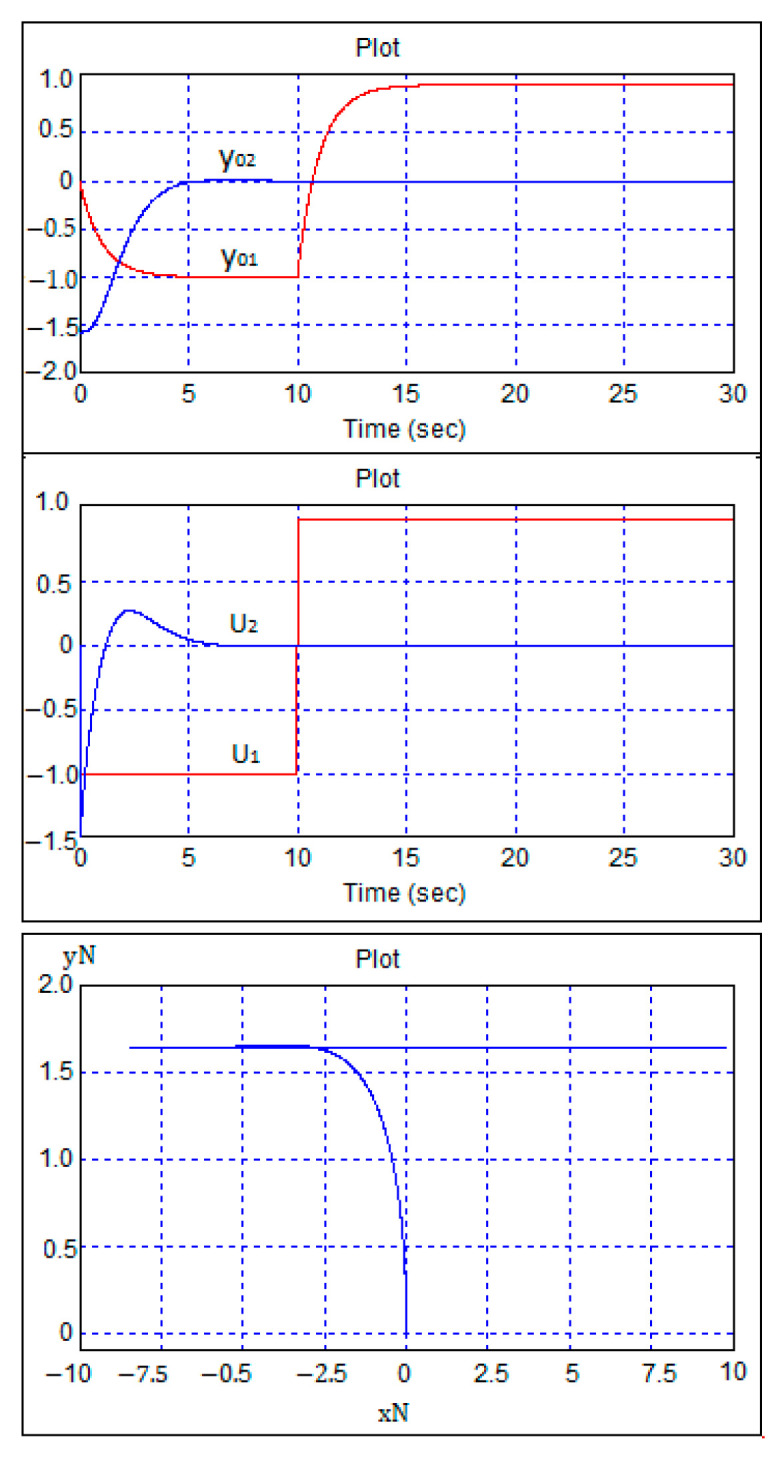
The robot maneuvers when moving back and forth.

As can be seen from Figure 13, a distance of about 2.26 m is required to turn the robot. Figure 14 illustrates the robot’s maneuvers when moving back and forth.

According to Figure 14, at zero time the robot stands at a point with coordinates (0, 0) with a heading of $-\frac{\pi }{2}$. Then, tasks are given to move backwards at a speed of 1 m/s and a heading of 0 radians. The robot leaves the starting point in reverse and, at the same time, turns clockwise, taking a horizontal position. Ten seconds after the start of movement, the speed command changes to forward movement parallel to the horizontal axis. 

**Figure 15:**
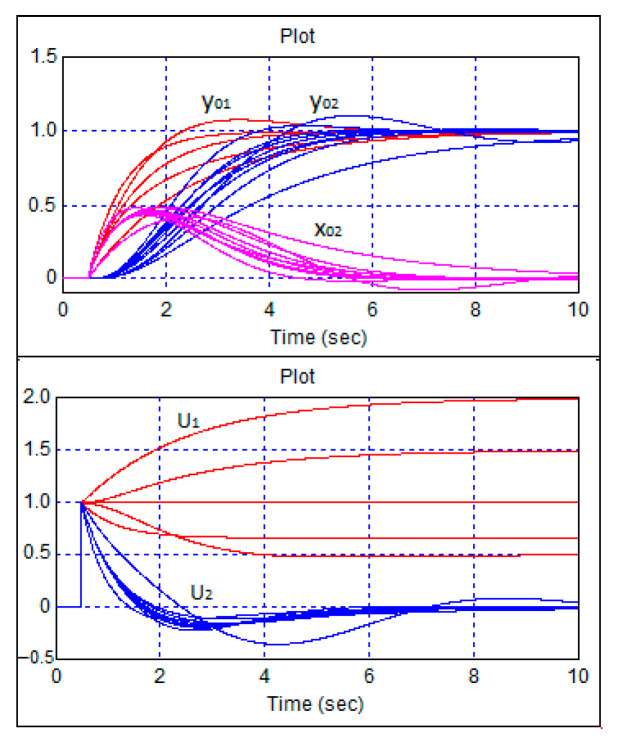
Transient processes in the robot control system when sequentially changing the coefficients $k_{1}$ … $k_{4}$ of the robot’s mathematical model by ±50% relative to their calculated values while tuning the regulator to the calculated values.

The results of the study on robust stability using a computer model of the system involved analyzing the transient processes in the control system when sequentially changing all the coefficients of the robot’s mathematical model by ±50% relative to their calculated values while tuning the regulator to the calculated values. These are presented in Figure 15.

Figure 15 shows that with significantly different values of the coefficients of the robot’s mathematical model, the transient processes converge to the desired value with zero steady-state error, indicating the stability of the control system. Thus, the simulation results convincingly demonstrate the stability of the control system. However, additional research is required to theoretically prove the stability of the closed-loop system, which is currently being conducted using Lyapunov’s second method.

**Figure 16:**
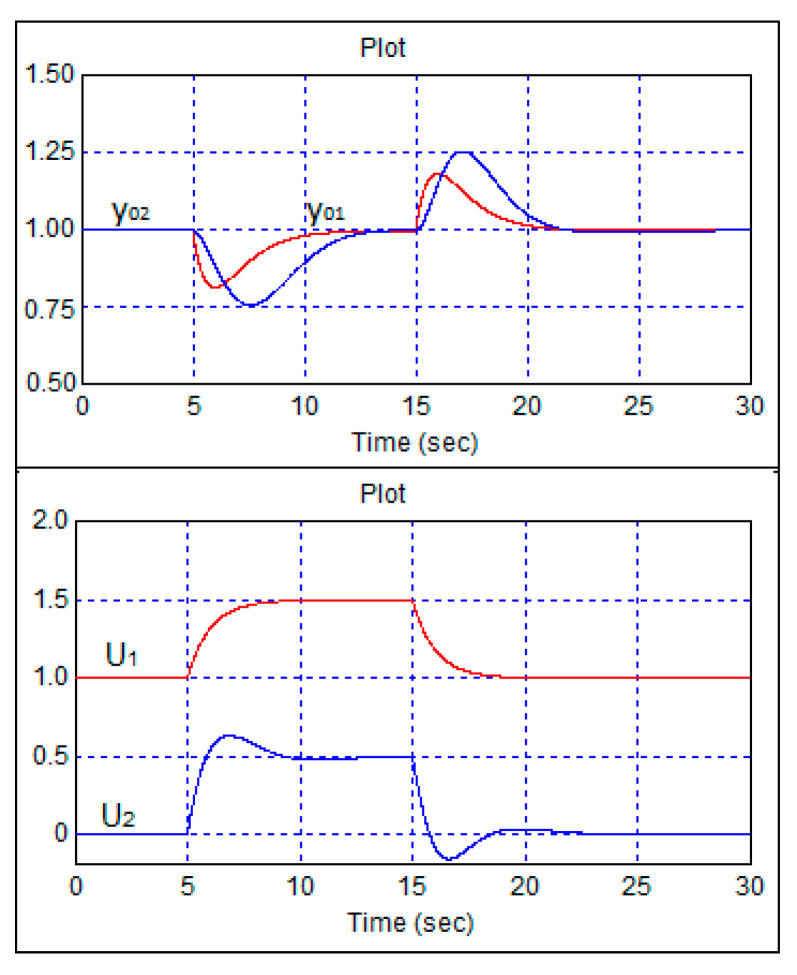
Transient processes in the robot control system at a nominal speed of 1 m/s and a course of 1 radian after 5 s and under the influence of disturbances.

A study of the impact of disturbances on the robot’s movement was carried out through computer simulation of the additional load on the drive and steering wheel motors. The results are presented in Figure 16.

Figure 16 shows the transient process where, at a nominal speed of 1 m/s and a course of 1 radian, an additional step load with an amplitude of 0.5 units was applied to the drive and steering wheel motors after 5 s, and the load was removed after 15 s. This load simulated movement uphill, wind load, changes in motor supply voltage, and other disturbances. The results presented in Figure 16 demonstrate the high quality of uncertainness suppression inherent in the proposed method.

As a summary, the main results of computer modeling of the control system of a four-wheeled robot are as follows:

- The control system consisting of a mathematical model and a controller demonstrated the possibility of achieving aperiodic transient processes that provided moderate amplitudes of control actions and stable movement of the robot;
- The use of a reference filter with certain coefficients helped to achieve the desired transient response and stability of the control system;
- The implementation of integrators with saturation into the model of the installation and controller made it possible to effectively compensate disturbances such as variable loads, changes in the mass of the robot, uneven paths, and voltage fluctuations in motor drives;
- The control algorithm ensured stable control of the robot both when moving forward and backward, demonstrating its versatility and adaptability to various motion scenarios;
- The control system’s ability to cope with delayed changes in speed and heading was demonstrated, highlighting its reliability and ability to handle dynamic scenarios;
- The influence of restrictions of the front wheels’ rotation on the control system was studied, showing that such restrictions, although they might delay transient processes, did not significantly affect the overall quality of the control;
- The results of computer simulation also illustrated the movement of the robot in fixed coordinates, demonstrating its ability to accurately follow given trajectories.

The proposed method focuses on detailed compensation of external influences, providing analytical tools for robot control in environments that require high precision and reliability. In contrast to previously described approaches that used neural network algorithms for adaptation in uncertain conditions [17] or focused on motion stability and Kalman filtering [21,23,46], this model was simplified and focused on compensation of impacts for single robots. We managed to achieve high control accuracy without taking into account complex kinematics—as was achieved in papers [15,22,47], while in papers [16,48,49], kinematics was taken into account with a focus on the relationship between angular and linear speeds depending on the orientation of the robot—which made it difficult to accurately follow a given trajectory due to angular and nonlinear limitations of the system; the interactions of the wheels with the surface were not taken into account, which, in real conditions, could lead to errors in control and movement trajectories. The method proposed in paper [50], although it can provide higher accuracy in controlled environments (as evidenced by lower mean squared errors in simulations), however, may not adapt quickly to changes in system dynamics without re-optimization. The approach presented in paper [51] might be more vulnerable to rapid or unexpected changes in robot dynamics, while our model could serve as a promising basis for developing control algorithms for four-wheeled mobile robots to quickly adapt to dynamic changes.

Thus, computer simulation analysis demonstrates the effectiveness of the developed control system for a four-wheeled robot. The system shows promising stability, adaptability to different scenarios, and the ability to cope with interference, making it suitable for practical applications in robotics. Further development and implementation of the algorithm on a microcontroller can increase its practical usefulness and contribute to the development of control systems for mobile robots.

The use of the presented method compensating for the object dynamics and disturbances allows us to create a new two-channel control algorithm. Thanks to this method, we were able to perform analytical synthesis of multi-channel linear control systems based on physically clear source data. It provided channel decoupling, the required closed-loop system dynamics, and zero static control error. It is important to note that the initial data were specified in the form of reference filters for a closed-loop system. This article presents a step-by-step procedure to facilitate the synthesis of a control algorithm. It allows us to determine some parameters of the reference filter considering the physical meaning of the control problem.

The developed algorithm ensures automatic stabilization of the speed and course of the robot when moving forward and backward. This significantly expands the capabilities of maneuvering and planning movement trajectories, which generates stabilization of the speed and course of the mobile robot. As the presented simulation results show, the proposed new control method allows the controller to successfully adapt and compensate for changes in the dynamics of the control object, even with significant changes in model parameters. Thus, changes in model parameters do not lead to alterations in the operational characteristics of the controller, such as inertia and the duration of transient processes. This clearly demonstrates the advantages of the proposed method over model-based techniques, where changes in model parameters require re-tuning of the controller parameters.

## 6. Further Perspectives

The practical application possibilities of the developed control algorithm include the use as the first stage of a general robot motion control system. It could have several advantages. First, digital algorithms can provide fast and accurate processing of sensor data, which is important for effective traffic control. Secondly, the digital algorithm can be easily customized and expandable, which allows for the adaptation of the control system to different conditions and requirements. It is planned to develop a digital version on an onboard microcontroller. To successfully implement it, careful development and testing are necessary. This includes algorithm design, microcontroller programming, integration with other control system components, and testing on a real robot.

Currently, theoretical studies on the stability of the closed-loop system are being conducted using Lyapunov’s second method.

Further research prospects include the integration of an inertial navigation system and an automatic control system for a mobile robot into a single system that uses machine learning methods and data from MEMS (microelectromechanical system) inertial sensors as feedback signals for the control and navigation of the robot. An analysis of approaches and methods for processing real-time sensor and control information using machine learning, as well as successful cases of machine learning application in the synthesis of a robot’s sensor and control systems, can be found in the article of Kondratenko et al. [52]. In our research, we planned to be the first to practically implement the idea of combining an inertial navigation subsystem built according to the “model-free” scheme and a subsystem based on the mathematical model of measured relations into one system to further conduct experiments to determine the class of the trajectory fragment as a spatial curve, according to inertia sensors’ data taken online within this fragment. Machine learning will be carried out using the data archives of the control system for different specified trajectories. The input data for learning will be calculated as the difference between the real and the given trajectories of the four-wheeled mobile robot.

## 7. Conclusions

A new inverse model-based control synthesis method for nonlinear objects has been developed. The main contribution of this work is a new approach to the problem of disturbance estimation and compensation, based on the new concept of reference filters. The method was applied to the problem of developing a controller for a four-wheeled mobile robot. An assessment of additive disturbances to the variables of the control object was carried out and, after filtering, these estimates were subtracted from the corresponding variables of the inverse model. Thanks to this approach, it was possible to develop a procedure for the structural synthesis of a control algorithm for a multi-channel nonlinear non-stationary object, while information about the output variables and state variables of the object was necessary. The results of the simulation of a closed-loop control system under various external influences demonstrated the speed, stability, and robustness of the new control system for a four-wheeled mobile robot with steering, that is, the effectiveness of the new control algorithm, and served as a proof of concept for the successful application of the promising, but so far incompletely researched, method of inverse models to robotics problems. We hope that this research will generate interest towards the inverse model method, especially its practical applications in the control of wheeled robots. This work is important for the development of autonomous robots and ensuring their reliable and accurate controls.

## Data Availability

The data presented in this study are available upon request from the corresponding authors.
